# Distribution of Primed T Cells and Antigen-Loaded Antigen Presenting Cells Following Intranasal Immunization in Mice

**DOI:** 10.1371/journal.pone.0019346

**Published:** 2011-04-29

**Authors:** Annalisa Ciabattini, Elena Pettini, Fabio Fiorino, Gennaro Prota, Gianni Pozzi, Donata Medaglini

**Affiliations:** Laboratorio di Microbiologia Molecolare e Biotecnologia (LA.M.M.B.), Dipartimento di Biotecnologie, Università di Siena, Siena, Italy; Centre de Recherche Public de la Santé (CRP-Santé), Luxembourg

## Abstract

Priming of T cells is a key event in vaccination, since it bears a decisive influence on the type and magnitude of the immune response. T-cell priming after mucosal immunization via the nasal route was studied by investigating the distribution of antigen-loaded antigen presenting cells (APCs) and primed antigen-specific T cells. Nasal immunization studies were conducted using the model protein antigen ovalbumin (OVA) plus CpG oligodeoxynucleotide adjuvant. Trafficking of antigen-specific primed T cells was analyzed *in vivo* after adoptive transfer of OVA-specific transgenic T cells in the presence or absence of fingolimod, a drug that causes lymphocytes sequestration within lymph nodes. Antigen-loaded APCs were observed in mediastinal lymph nodes, draining the respiratory tract, but not in distal lymph nodes. Antigen-specific proliferating T cells were first observed within draining lymph nodes, and later in distal iliac and mesenteric lymph nodes and in the spleen. The presence at distal sites was due to migration of locally primed T cells as shown by fingolimod treatment that caused a drastic reduction of proliferated T cells in non-draining lymph nodes and an accumulation of extensively divided T cells within draining lymph nodes. Homing of nasally primed T cells in distal iliac lymph nodes was CD62L-dependent, while entry into mesenteric lymph nodes depended on both CD62L and α4β7, as shown by *in vivo* antibody-mediated inhibition of T-cell trafficking. These data, elucidating the trafficking of antigen-specific primed T cells to non-draining peripheral and mucosa-associated lymph nodes following nasal immunization, provide relevant insights for the design of vaccination strategies based on mucosal priming.

## Introduction

Mucosal T-cell priming is a critical early event that deeply influences the type and magnitude of the immune response to local vaccination. Mucosal inductive sites are constituted by organized mucosa-associated lymphoid tissues (MALT) as well as local mucosa-draining lymph nodes, where antigens (Ag) are taken up, and B- and T-cell responses are induced [1]. The pattern of signals received during the initial interactions between naïve CD4^+^ T cells and antigen presenting cells (APCs) determines T-helper activation and differentiation in cells that are able to interact with cognate B cells, regulating multiple stages of their immune function [2]. T-cell priming indeed influences both B- and T-cell memory generation, thus determining the success of a vaccination strategy [3], [4]. Recent studies have shown that the frequency of Ag-specific primed CD4^+^ T cells can predict the intensity of the secondary humoral responses [5].

Crucial events in T-cell priming following mucosal vaccination, including mechanisms of local Ag-uptake, APCs recruitment and mobilization, as well as redistribution of primed T cells within lymphoid compartments, need to be carefully elucidated to inform the rational design of vaccination strategies. Dendritic cells (DCs) play a major role in the immune surveillance of the mucosal surfaces and in the initiation of immune responses. Upon encounter with foreign antigens, DCs migrate from mucosa to the nearest inductive site where they act as APCs [6]. Primary T-cell response results in expansion of naïve Ag-specific T cells, with generation of a pool of memory Ag-specific T lymphocytes [7]. The study of mucosal immune responses has been mainly focused on the characterization of effector humoral responses [8]–[10], and only recently mucosal T-cell priming is beginning to be elucidated [11]–[14].

We have previously shown that after mucosal priming, Ag-specific proliferated T cells are present also in distal non-draining lymphoid compartments [15], [16]. This can be due to a dissemination of Ag-loaded DCs towards non-draining lymph nodes and subsequent proliferation of resident T cells, or to a redistribution of T cells primed in the lymphoid compartment draining the immunization site.

In the present work we investigated the distribution of Ag-loaded APCs, the subsequent CD4^+^ and CD8^+^ T-cell priming and trafficking towards distal lymphoid sites after nasal immunization with a model vaccine formulation constituted by ovalbumin (OVA) plus CpG oligodeoxynuclotide (ODN) 1826 as adjuvant. By using fluorescent OVA, we analyzed the distribution of Ag-loaded APCs at different time points within draining and distal lymph nodes and spleen. The trafficking of primed CD4^+^ and CD8^+^ T cells was studied in mice adoptively transferred with TCR transgenic T cells [17]. *In vivo* treatment with fingolimod (FTY720), a drug that causes sequestration of T cells in lymph nodes [18], was used to further characterize T-cell redistribution to Ag-free lymph nodes. The role of migration molecules, such as CD62L and α4β7, in homing of T cells primed by nasal immunization to different lymphoid compartments was also dissected using *in vivo* antibody blocking assays.

## Results

### 1. Ag-bearing APCs localization after nasal immunization

**Figure 1:**
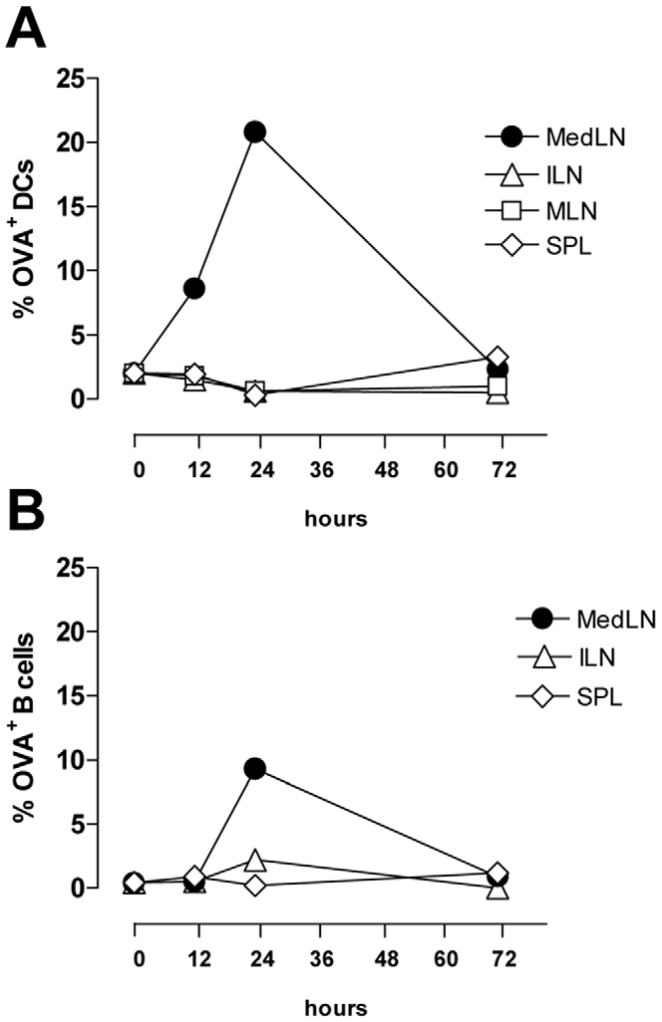
Time-course analysis of antigen-loaded APCs localization. C57BL/6 mice were immunized by the nasal route with OVA-Alexa fluor 647 conjugate (25 µg/mouse) and CpG ODN (20 µg/mouse). At different time points (0, 12, 24 and 72 h), DCs and B cells were isolated from draining (mediastinal [MedLN]) and distal (iliac [ILN], mesenteric [MLN]) lymph nodes and spleen (SPL). The percentages of DCs (A) and B cells (B) positive for fluorescent OVA were analysed by flow cytometry. Shown is the percentage of OVA-Alexa fluor 647 positive cells among total live CD11c^+^ MHC class II^+^ cells purified using anti CD11c-conjugated magnetic beads (A) or B220^+^ cells (B).

We have previously observed that after nasal immunization, primed T cells are present not only in draining lymph nodes but also in distal lymphoid organs [15], [16]. This can be due to a dissemination of Ag-bearing DCs that leads to a local proliferation of resident naïve T cells or a redistribution of primed T cells from the lymphoid compartment draining the immunization site. To study the fate of Ag-bearing APCs after mucosal administration, OVA-Alexa fluor 647 conjugate was inoculated with the mucosal adjuvant CpG ODN by the nasal route, and the presence of Ag-loaded DCs and B cells was studied at different time points, in both draining and distal lymph nodes and spleen. Following nasal administration, Ag-loaded DCs (CD11c^+^ MHC class II^+^) and B cells were observed mainly in mediastinal lymph nodes (Fig. 1), with about 9% of OVA^+^ DCs after 12 h, and 21% after 24 h (Fig. 1 A). OVA^+^ B cells were also observed in mediastinal lymph nodes 24 h after immunization with about 10% of positive cells (Fig. 1 B). Ag-loaded DCs and B cells declined 72 h after Ag administration (Fig. 1). In distal lymphoid organs, OVA^+^ DCs and B cells were not detected at any time point (Fig. 1). These data indicate that following nasal immunization Ag-loaded APCs are localized in mediastinal lymph nodes that drain the respiratory tract, and do not disseminate towards distal lymphoid sites.

### 2. Ag-specific T-cell proliferation and dissemination into distal lymphoid organs

**Figure 2:**
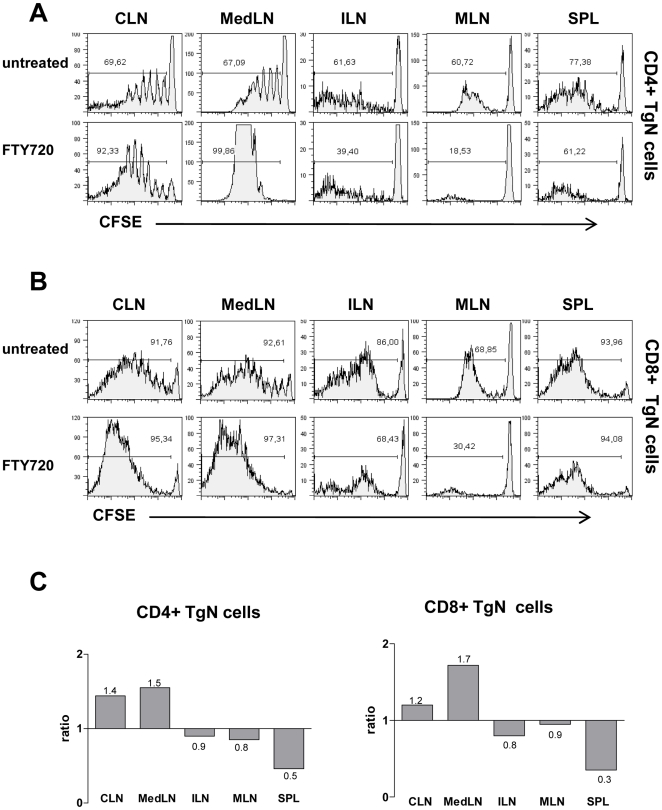
Effect of treatment with FTY720 on dissemination of primed T cells after nasal immunization with OVA plus CpG ODN. CD4^+^ and CD8^+^ T cells, isolated from OT-II and OT-I mice, were labelled with CFSE and adoptively transferred into recipient C57BL/6 Ly5.1 mice. Twenty-four hours later, recipient mice were immunized with OVA (25 µg/mouse) and CpG ODN (20 µg/mouse) by the nasal route. FTY720 was administered i.p. 18 h after immunization and every 24 h until harvest 5 days after immunization. **A–B**. CD4^+^ (A) and CD8^+^ (B) TgN T- cell proliferation assessed in cervical (CLN), mediastinal (MedLN), iliac (ILN), and mesenteric (MLN) lymph nodes, and spleen (SPL), by CFSE dilution. Histograms are gated on CD4^+^ CD45.2^+^ (A) or CD8^+^ CD45.2^+^ (B) population, with light scatter properties of lymphocytes. Results are representative of three independent experiments performed with three mice per group. Reported values indicate the percentage of dividing OVA-specific TgN T cells. **C.** Ratio between the absolute number of OVA-specific CD4^+^ and CD8^+^ TgN T cells detected in FTY720- treated *versus* untreated mice. Values of ratios for each organ are reported.

The lack of migration of Ag-bearing APCs towards distal lymphoid organs following nasal immunization suggests that T cells, primed within the mucosal inductive site, disseminate towards distal lymph nodes. To demonstrate this, OVA-specific transgenic (TgN) CD4^+^ and CD8^+^ T cells, labeled with CFSE, were adoptively transferred into recipient mice that were immunized with the model formulation OVA and CpG ODN by the nasal route. Experiments were performed in the presence or absence of FTY720, an analog of sphingosine 1-phosphate that inhibits T lymphocyte egress from lymph nodes [18]. Since FTY720 can affect also DCs migration [19], [20], we started the drug administration 18 hours after nasal immunization, in order to allow respiratory DCs to migrate from airway to draining lymph nodes and initiate Ag-presentation to resident T cells. The OVA-specific clonal expansion was assessed in draining and distal lymph nodes 5 days after nasal immunization, since we previously demonstrated the presence of proliferated T cells within non draining sites at this time point [15], [16]. Mice nasally immunized with OVA and CpG ODN in the absence of FTY720 showed an Ag-specific clonal expansion of both CD4^+^ and CD8^+^ TgN cells in cervical and mediastinal lymph nodes that act as draining lymph nodes following intranasal immunization [21], and also in distal iliac and mesenteric lymph nodes, and in the spleen (Fig. 2 A and B). While a continuous division profile of T cells was observed in draining lymph nodes with about 6 division peaks, early cell generations were absent in non-draining lymph nodes (Fig. 2 A and B). The treatment with FTY720 determined an accumulation of more extensively divided T cells in draining cervical and mediastinal lymph nodes with about 92% of proliferating lymphocytes respect to 69% of untreated mice (Fig. 2 A). On the contrary, in distal lymph nodes the presence of proliferating T cells was drastically reduced by FTY720 treatment with about 40% and 19% of dividing cells in iliac and mesenteric lymph nodes respectively, compared to about 60% observed in untreated mice (Fig. 2 A). The same effect of FTY720 treatment was observed for CD8^+^ TgN T cells, both in draining and distal lymph nodes (Fig. 2 B). Furthermore, the higher percentages of proliferating T cells observed in cervical and mediastinal lymph nodes of FTY720-treated *versus* untreated mice, corresponded to a higher absolute number of TgN CD4^+^ and CD8^+^ T cells present in the draining lymph nodes, thus confirming the sequestration of lymphocytes within the priming sites. Indeed, as shown in figure 2 C, the ratio between the amount of CD4^+^ or CD8^+^ TgN T cells measured in FTY720-treated *versus* untreated mice was >1 in draining sites and <1 in distal lymphoid organs, since the clonal expanded T cells could not disseminate due to the fingolimod effect. Also in the spleen the number of TgN T cells was reduced by FTY720 treatment (Fig. 2 C), even if no significant difference in the percentage of proliferating CD4^+^ and CD8^+^ T cells was observed (Fig. 2 A and B) .

Taken together these data demonstrate that after nasal immunization, antigen-specific CD4^+^ and CD8^+^ T cells are primed in the regional mucosa-draining lymph nodes and then became able to leave the priming compartment and traffic towards distal non-draining lymph nodes and spleen.

### 3. Migration molecules involved in dissemination of nasally primed T cells towards distal lymphoid compartments

**Figure 3:**
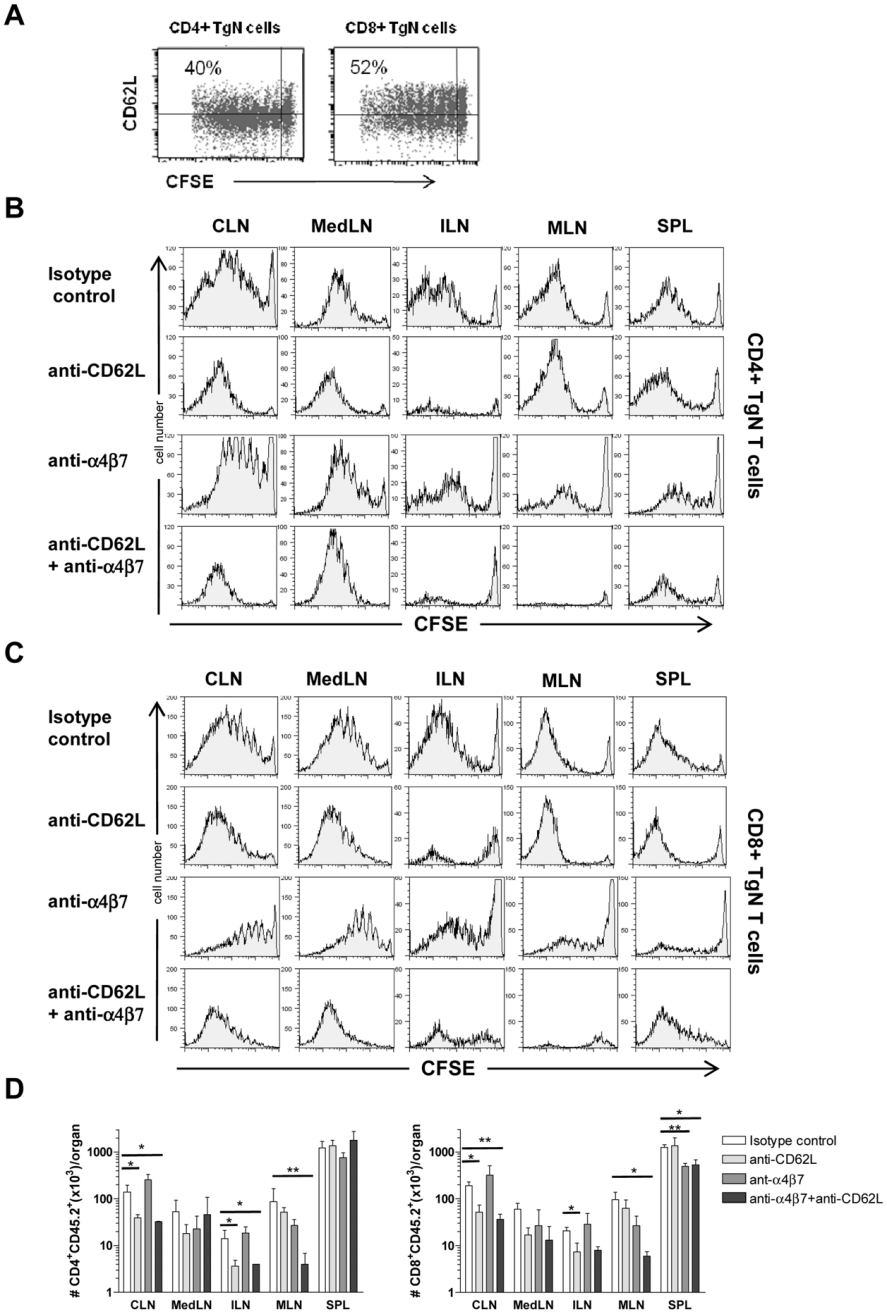
Role of CD62L and α4β7 on dissemination of primed T cells. Adoptively transferred mice were nasally immunized with OVA (100 µg/mouse) and CpG ODN (20 µg/mouse) and treated i.v. with anti-CD62L, anti-α4β7, anti-CD62L plus anti-α4β7, or isotype control antibodies beginning 6 h after immunization, and continuing every 24 h until harvest 5 days after immunization. **A**. Dot plot analysis of CD62L *versus* CFSE of CD4^+^ and CD8^+^ TgN cells collected from cervical lymph nodes of mice nasally immunized and treated with isotype control antibody. Values indicate the percentage of proliferating TgN T cells that express CD62L. **B–C**. OVA-specific proliferation of CD4^+^ (B) and CD8^+^ (C) TgN T cells following treatment with isotype control, anti-CD62L, anti-α4β7 or anti-CD62L plus anti-α4β7 antibodies, analysed in cervical (CLN), mediastinal (MedLN), iliac (ILN) and mesenteric (MLN) lymph nodes and spleen (SPL). Histograms are gated on CD4^+^ CD45.2^+^ (A) or CD8^+^ CD45.2^+^ (B) population, with light scatter properties of lymphocytes. **D**. CD4^+^ and CD8^+^ TgN T cell absolute number detected in lymph nodes and spleens of mice treated with anti-CD62L (light grey bar), anti-α4β7 (grey bar), anti-CD62L plus anti-α4β7 (black bar), or isotype control (open bar) antibodies. Values are reported as mean±standard error of the mean (SEM) of three mice per group.* *P*≤0.05, ** *P*≤0.01 *versus* isotype control.

The dissemination towards non-draining lymphoid sites of T cells primed by nasal route was further characterized by identifying the molecules involved in distal lymph nodes entry. Naïve and central memory T cells express CD62L, a molecule that regulates lymphocyte entry into peripheral lymph nodes by mediating the leukocyte rolling on blood vessel endothelium [22], [23]. After nasal immunization with OVA and CpG ODN, about 40% of CD4^+^ and 50% of CD8^+^ T cells were CD62L-positive in cervical lymph nodes (Fig. 3 A). To establish the role of CD62L in homing of primed T lymphocytes into distal lymphoid organs following nasal immunization, groups of mice immunized with OVA and CpG ODN were treated with anti-CD62L or isotype control antibodies beginning 6 h after immunization, and continuing every 24 h until day 5. The treatment with anti-CD62L dramatically reduced the presence of CD4^+^ and CD8^+^ TgN T cells within distal iliac lymph nodes, while did not prevent the T-cell entry into mesenteric lymph nodes or spleens (Fig. 3 B and C). The absolute number of CD4^+^ and CD8^+^ TgN T cells detected in draining lymph nodes of mice treated with anti-CD62L antibodies was lower than isotype control-treated mice (Fig. 3 D), thus demonstrating that the early treatment with anti-CD62L antibody blocks the recruitment of naïve Ag-specific T cells within the draining lymph nodes. Also in distal lymph nodes the number of T cells in mice treated with anti-CD62L antibodies was lower than control mice (Fig. 3 D). T cells primed in cervical and mediastinal lymph nodes were therefore allowed to leave the draining site, but not to re-enter in distal iliac lymph nodes, due to the blocking of L-selectin. On the contrary, only a slight reduction in the amount of primed T cells was observed in mesenteric lymph nodes following anti-CD62L treatment (Fig. 3 D), where primed T cells were still allowed to enter (Fig. 3 B and C); this can be explained by the role played by α4β7 molecule that regulates the lymphocyte entry in mesenteric lymph nodes [24], [25]. This integrin was indeed expressed by more than 25% of TgN cells collected from mesenteric lymph nodes in control mice (data not shown). To further characterise the role of α4β7 in homing of primed T lymphocytes into distal lymphoid organs, groups of mice nasally immunized with OVA and CpG ODN were daily treated with anti-α4β7 or with anti-α4β7 plus anti-CD62L. The treatment with anti-α4β7 antibody was able to reduce, but not to completely block, the entry of primed CD4^+^ and CD8^+^ TgN T cells within mesenteric lymph nodes (Fig. 3 B and C). The total blockage of primed T-cell entry within mesenteric lymph nodes was obtained by administering anti-α4β7 and anti-CD62L antibodies together (Fig. 3 B and C). In the spleen we did not observe a decrease of primed T cells following anti-CD62L antibody treatment, while a significant reduction of migrated T cells was reported after anti-α4β7 administration (Fig. 3 D). Collectively, these results indicate that CD62L plays a critical role in homing of nasally primed T lymphocytes in iliac lymph nodes while the entrance of primed T cells within mesenteric lymph nodes is dependent on both CD62L and α4β7.

## Discussion

In this work we show that T cells - and not antigen-loaded APCs - primed by the nasal route with OVA and CpG ODN migrate to distal lymph nodes and to the spleen, and that the entry of nasally primed T cells into iliac lymph nodes is strictly CD62L-dependent, while homing to mesenteric lymph nodes is regulated by both CD62L and α4β7.

We have previously observed that divided T cells, primed by nasal immunization, are detectable in distal lymphoid organs, such as iliac and mesenteric lymph nodes and in the spleen [15], [16] and we hypothesized that this could be due to the dissemination of Ag-bearing APCs into distal lymphoid sites, or to the redistribution of T cells primed in the draining lymph nodes. It is commonly accepted, mostly from Langerhans cells studies [26], that Ag-loaded APCs terminally migrate to draining lymph nodes where they present the antigen to cognate T and B cells residing within the lymphoid organ [27]. However, it has been recently shown that selected adjuvant molecules are able to alter the migratory properties of DCs leaving the vaccination site, allowing a fraction of these mobilized DCs to evade sequestration within draining lymph nodes and localize in other secondary lymphoid organs [28]. Here, we clearly demonstrated that upon nasal immunization with OVA and CpG ODN, OVA-bearing DCs and B cells were observed only in draining lymph nodes, and not in any distal lymph nodes or in the spleen. This is in line with what observed after nasal instillation of the fluorescent dye CFSE, that showed an accumulation of fluorescent DCs predominantly within draining lymph nodes but not in other lymphoid organs [29], [30]. It has also been shown that CpG adjuvant is unable to alter the migratory properties of DCs after injection in the footpad [31].

The redistribution of primed CD4^+^ and CD8^+^ T cells in distal lymphoid sites was demonstrated by treating mice with FTY720. Mice treated with FTY720 showed a higher accumulation of more extensively divided T cells within draining (cervical and mediastinal) lymph nodes, while in distal lymph nodes the percentage of divided TgN cells was largely reduced. The sequestration of lymphocytes within the priming sites was also confirmed by the higher count of TgN T cells retained in draining lymph nodes of FTY720-treated mice compared to control. These data show that following nasal immunization with OVA and CpG, priming of T lymphocytes occurs in the draining lymph nodes and then activated cells disseminate towards non-draining lymphoid compartments. This is in line with what was recently reported following systemic [32], oral [33] and sublingual [34] immunization. The results reported in this study obtained using CpG ODN as adjuvant, were comparable with what observed using different adjuvants, (i.e. cholera toxin B subunit and alpha-galactosylceramide; data not shown), suggesting that our observations do not depend on the specific adjuvant employed. These data, showing the expansion and dissemination of antigen-specific primed T cells following intranasal vaccination, demonstrate that the mucosal route of immunization via the respiratory tract can be very efficient in vaccine priming. These findings provide the conceptual basis for the development of combined mucosal/parenteral prime-boost strategies capable of eliciting improved immune responses than either route alone [35],[36].

Migration of lymphoid cells throughout the body is regulated by the expression of multiple adhesion molecules and chemokine receptors that interact with corresponding ligands on endothelial and stromal cells. T-cell access to peripheral lymph nodes is mediated by the expression of molecules such as CD62L that controls rolling events on blood vessel endothelium by interacting with peripheral lymph node addressin (PNAd) [22], [23]. CD62L is well expressed on naïve and central memory T cells, while is down regulated on effector cells. Another molecule that plays an important role in the recruitment of circulating lymphocytes to the immune system of the gastrointestinal tract is the integrin α4β7 [24], [25] which binds to mucosal addressin cell adhesion molecule-1 (MAdCAM-1), a ligand preferentially expressed on high endothelial venules of Peyer's Patches, mesenteric lymph nodes and intestinal lamina propria [37]. Here we provide evidence that after nasal immunization with OVA and CpG ODN, about 40% of CD4^+^ and 50% of CD8^+^ primed T lymphocytes present in draining lymph nodes still expressed CD62L, thus suggesting that these cells use this molecule to disseminate towards distal lymph nodes. To demonstrate the role of CD62L and α4β7 in the redistribution of nasally primed T cells in peripheral and mucosa-associated lymph nodes, we treated mice with anti-CD62L or anti-α4β7 antibodies. After blocking CD62L molecule, the migration of activated T cells into iliac lymph nodes was markedly inhibited, while the entry of cells into the spleen and mesenteric lymph nodes was not prevented. The absolute number of TgN T cells in iliac lymph nodes of mice treated with anti-CD62L antibodies was lower compared to control mice, thus confirming the block of entry. The anti-CD62L antibody treatment shows that primed T cells in cervical and mediastinal lymph nodes are allowed to leave the draining site, but not to re-enter in distal iliac lymph nodes, demonstrating that the dissemination of primed T cells in the iliac lymph nodes is mediated by the expression of CD62L. In the spleen we did not observe a decrease of primed T cells following antibody treatment, since CD62L is not involved into spleen homing [38]. We also observed that CD62L plays a critical role in the continuous recruitment of naïve OVA-specific T cells within draining lymph nodes during the priming event, as shown by the reduced counts of CD4^+^ and CD8^+^ TgN T cells and the different profiles of dividing cells in antibody-treated compared to control mice. As reported by Jenkins and colleagues [39], after parenteral immunization about a half of Ag-specific T cells inside the draining lymph nodes are late-arriving cells, and their progeny still express CD62L consistent with a memory phenotype.

The entry of nasally primed T cells into mesenteric lymph nodes was inhibited by neither anti-CD62L nor anti-α4β7 antibody treatment alone, but only by administration of both antibodies, demonstrating regulation by both CD62L and α4β7. Thus, here we show that CD62L and α4β7 promote entry into mesenteric lymph nodes of nasally primed T cells and not only of naïve T cells as previously reported [40], [41]. Interestingly, the treatment with anti-α4β7 altered also the migration of primed T cells into the spleen, as shown by the lower absolute number of TgN T cells, especially cytotoxic lymphocytes, compared to control mice. It has been shown that MAdCAM-1 is highly expressed on sinus lining cells closest to the lymphoid white pulp of the spleen [42], and this receptor-ligand interaction can be responsible for the observed migration of proliferated T cells into the spleen.

These data, elucidating the dissemination of primed T cells to distal lymphoid organs following nasal immunization, expand the knowledge on functioning of the mucosal immune system and have important implications for rational design of optimised vaccination strategies and prime-boost schemes.

## Methods

### Ethics Statement

Mice were housed in the animal facilities at the University of Siena (Decreto n° 146 2005-A) and treated according to national guidelines (Decreto Legislativo 27/01/1992 n° 116, implementing 86/609/CEE Directive). All animal studies were approved by the Ethics Committee “Comitato Etico Locale dell'Azienda Ospedaliera Universitaria Senese” (protocol number 22933).

### Mice

Nine-weeks old female OT-I [43] and OT-II [44] TCR-transgenic mice, C57BL/6 and C57BL/6-Ly5.1 mice were purchased from Charles River (Lecco, Italy). Transgenic mice were maintained under specific pathogen-free conditions.

### Nasal immunization with fluorescent OVA and DCs isolation

OVA uptake was tracked *in vivo* using Ovalbumin-Alexa fluor 647 conjugate (Invitrogen Molecular Probes, Eugene, OR, USA). The fluorescent OVA (25 µg/mouse) was mixed with the mucosal adjuvant CpG oligodeoxynuclotide (ODN) 1826 (Eurofins MWG Operon, Ebersberg, Germany) (20 µg/mouse) and administered by nasal route. C57BL/6 mice were lightly anaesthetized by intraperitoneal injection of tiletamine and zolazepam hydrochloride (Zoletil 20, Laboratoires Virbac, France, 6 mg/kg) and xylazine (Xilor 2%, Bio 98 Srl, Italy, 3 mg/kg), held in a vertical position and then inoculated drop wise into the nostrils in a total volume of 20 µl. Groups of four mice were sacrificed 0, 12, 24 and 72 h after nasal immunization. Mediastinal, iliac and mesenteric lymph nodes and spleens were harvested and digested in Click’s medium (Sigma-Aldrich) with 2 mg/ml of Collagenase D (Roche Applied Science, Penzberg, Germany) for 30 min at 37°C. Organs were mashed onto nylon screens (Sefar Italia, Torino, Italy) and washed in phosphate buffered saline (PBS) with 0.5% of bovine serum albumin (BSA, Sigma-Aldrich) and 2 mM EDTA (Mallinckrodt Baker, Philipsburg, NJ, USA). DCs were purified by magnetic activating cell sorting (MACS) using CD11c-conjugated microbeads (Miltenyi Biotec GmbH, Germany) according to manufacturer's instructions.

### Adoptive transfer of transgenic T cells

Single-cell suspensions from the spleen and pooled lymph nodes (cervical, brachial, axillary, mesenteric and iliac lymph nodes) of OT-I and OT-II transgenic mice were enriched for CD8^+^ and CD4^+^ T cells using the EasySep negative CD8^+^ and CD4^+^ T cell enrichment kit, respectively (StemCell Technologies, Vancouver, BC, Canada) according to the manufacturer's protocol. CD8^+^ and CD4^+^ T cells were stained with carboxy-fluorescein diacetate succinimidyl ester (CFSE, 7.5 µM, Invitrogen, USA) [45], for 10 min at 37°C. An amount of 5×10^6^ of mixed CFSE-labelled CD8^+^ and CD4^+^ T cells was injected into the tail vein of the recipient C57BL/6-Ly5.1 mice.

### FTY720 treatment and *in vivo* Ab-mediated blocking of migration molecules

Twenty-four hours after adoptive transfer of CFSE-labelled OT-I CD8^+^ and OT-II CD4^+^ T cells, recipient C57BL/6-Ly5.1 mice were immunized with 25 or 100 µg of OVA (Sigma-Aldrich) and 20 µg of CpG ODN administered by nasal route as described above. To prevent T-cell egress from lymph nodes, mice were injected i.p. with 1 mg/Kg body weight of FTY720 (Merck Chemicals, Nottingham, UK) in 100 µl of PBS 18 h after immunization and then every 24 h until harvest. In other experiments, purified anti-CD62L (MEL-14, Southern Biotechnology, USA), anti-α4β7 (DATK-32, eBioscience, USA) or rat IgG2a isotype control (eBioscience) were administered i.v. at 100 µg/mouse 6 h after immunization and then every 24 h until harvest. Five days after immunization, lymph nodes and spleens were collected and analysed by flow cytometry.

### Flow cytometric analysis

Single-cell suspensions from cervical, mediastinal, iliac and mesenteric lymph nodes and spleens were obtained as previously described [46]. Cells were incubated with Fc-blocking solution [0.5 mg CD16/CD32 mAb (clone 93) (eBioscience), 5% mouse serum, 5% rat serum, 0.2% sodium azide (all from Sigma-Aldrich) in 100 ml of HBSS] for 30 min on ice. Cells were stained with PerCP-conjugated anti-mouse CD4 (clone RM 4-5) or CD8 (clone 53-6.7) (BD Pharmingen), PE-conjugated anti-mouse CD45.2 (clone 104) and with APC-conjugated anti-mouse CD62L (Ly-22) (clone MEL-14) (all from eBioscience) for 30 min on ice. Analysis of T-cell clonal expansion was performed on single samples collected from each animal. The absolute number of transgenic T cells detected in each organ was determined by using CountBright absolute counting beads (Invitrogen Molecular Probes, USA) according to the manufacturer's protocol.

DCs present in the CD11c-positive fraction after MACS were labelled with FITC-conjugated I-A/I-E (clone M5/114.15.2), PE-conjugated CD11c (clone N418) (all from eBioscience), and propidium iodide (Sigma-Aldrich) for excluding dead cells. B cells present in the CD11c-negative fraction were labelled with PerCP-conjugated CD45R/B220 (clone RA3-6B2, BD Pharmingen). Samples were analyzed by flow cytometry (FACScalibur, Becton Dickinson, San Diego, CA). Data analysis was performed by using Flow Jo software (Treestar, Ashland, OR, USA).

### Statistical analysis

Statistical significance was assessed by Student's *t* test. A value of *P*≤0.05 was considered significant.
